# P-301. PrEP Use among Women of Reproductive-Age Enrolled in the Study of Treatment and Reproductive Outcomes (STAR)

**DOI:** 10.1093/ofid/ofaf695.521

**Published:** 2026-01-11

**Authors:** Erin R Carr, Madison S Meyer, Nicholas Fonseca Nogueira, Laura Beauchamps, Daniel Westreich, Seble G Kassaye, Aadia Rana, Deborah Konkle-Parker, Deborah Jones Weiss, Anandi N Sheth, Maria L Alcaide

**Affiliations:** University of Miami Miller School of Medicine, Miami Beach, Florida; University of Miami Miller School of Medicine, Miami Beach, Florida; Division of Infectious Diseases, Department of Medicine, University of Miami Miller School of Medicine, Parkland, Florida; University of Miami Miller School of Medicine, Miami Beach, Florida; UNC-Chapel Hill, Chapel Hill, North Carolina; Georgetown University Medical Center, Washington, DC, Washington, DC; University of Alabama-Birmingham School of Medicine, Birmingham, AL; University of Mississippi Medical Center, Jackson, MS; University of Miami Miller School of Medicine, Miami Beach, Florida; Emory University School of Medicine, Atlanta, Georgia; Division of Infectious Diseases, Department of Medicine, University of Miami Miller School of Medicine, Parkland, Florida

## Abstract

**Background:**

HIV prevention efforts in the US have primarily focused on men who have sex with men, limiting pre-exposure prophylaxis (PrEP) awareness and uptake among women. This study examines PrEP use among reproductive-age women without HIV (WWOH) enrolled in the Study of Treatment and Reproductive Outcomes (STAR), the largest cohort of reproductive-age women in the Southern US.

**Methods:**

STAR is a longitudinal cohort of reproductive-age WWOH (eligible if aged 18-45 years and risk factors for HIV) in six cities (Miami, FL; Atlanta, GA; Chapel Hill, NC; Washington, DC; Birmingham, AL; Jackson, MS). Demographics, medical history, STI/HIV history, and PrEP use were self-reported. STI/HIV were assessed using commercially available tests.

**Results:**

Among 362 WWOH eligible for PrEP, 36 (9.9%) reported ever using PrEP (24 current use and 12 prior use). Of those on PrEP who reported PrEP source, 6 obtained it through a clinical study, 12 from a healthcare provider, and 1 from non-medical sources. All participants who reported PrEP use had used tenofovir disoproxil/emtricitabine. Women who reported ever using PrEP were older (35 vs 31 years, p=0.001); the majority lived in Miami (53%, p=0.001); identified as non-Hispanic Black (66%, p=0.163); had an earlier sexual initiation (14.0 vs 15.8 years, p=0.004); reported more lifetime partners (34 vs 10, p=0.003); and had engaged in transactional sex in the prior 5 years (22% vs 10%, p=0.047). Though current STI rates (gonorrhea, chlamydia, syphilis, and trichomoniasis) did not differ by PrEP use, prior syphilis and chlamydia were higher among those who had ever used PrEP (19% vs 4.3%, p=0.002 and 58% vs 34%, p=0.007). Reasons to discontinue PrEP included medication cost (12, 100%), provider access (12, 100%), and periodic use (12, 100%).

**Conclusion:**

Despite high vulnerability to HIV among reproductive-age women in the Southern US, PrEP use was low in this sample, and discontinuation high. Affordable and accessible novel modes of PrEP delivery, and investigation on bundling HIV PrEP with DoxyPEP are urgently needed to promote access, uptake, and sustainment of PrEP use among young women.

**Disclosures:**

Daniel Westreich, PhD, Sanofi-Pasteur: Advisor/Consultant Maria L. Alcaide, MD, Gilead: Advisor/Consultant

